# Supplementary material for: Causal Relationship between Aging and Anorexia Nervosa: A White-Matter-Microstructure-Mediated Mendelian Randomization Analysis
Source: Biomedicines. 2024 Aug 16;12(8):1874. doi: 10.3390/biomedicines12081874 (PMC11351342; doi:10.3390/biomedicines12081874)
Supplement: Supplementary file 1 [file biomedicines-12-01874-s001.zip › Supplementary Figure/Figure S9/Leaveoneout plot of MR analysis for ubm-b-1726 to AN.pdf]

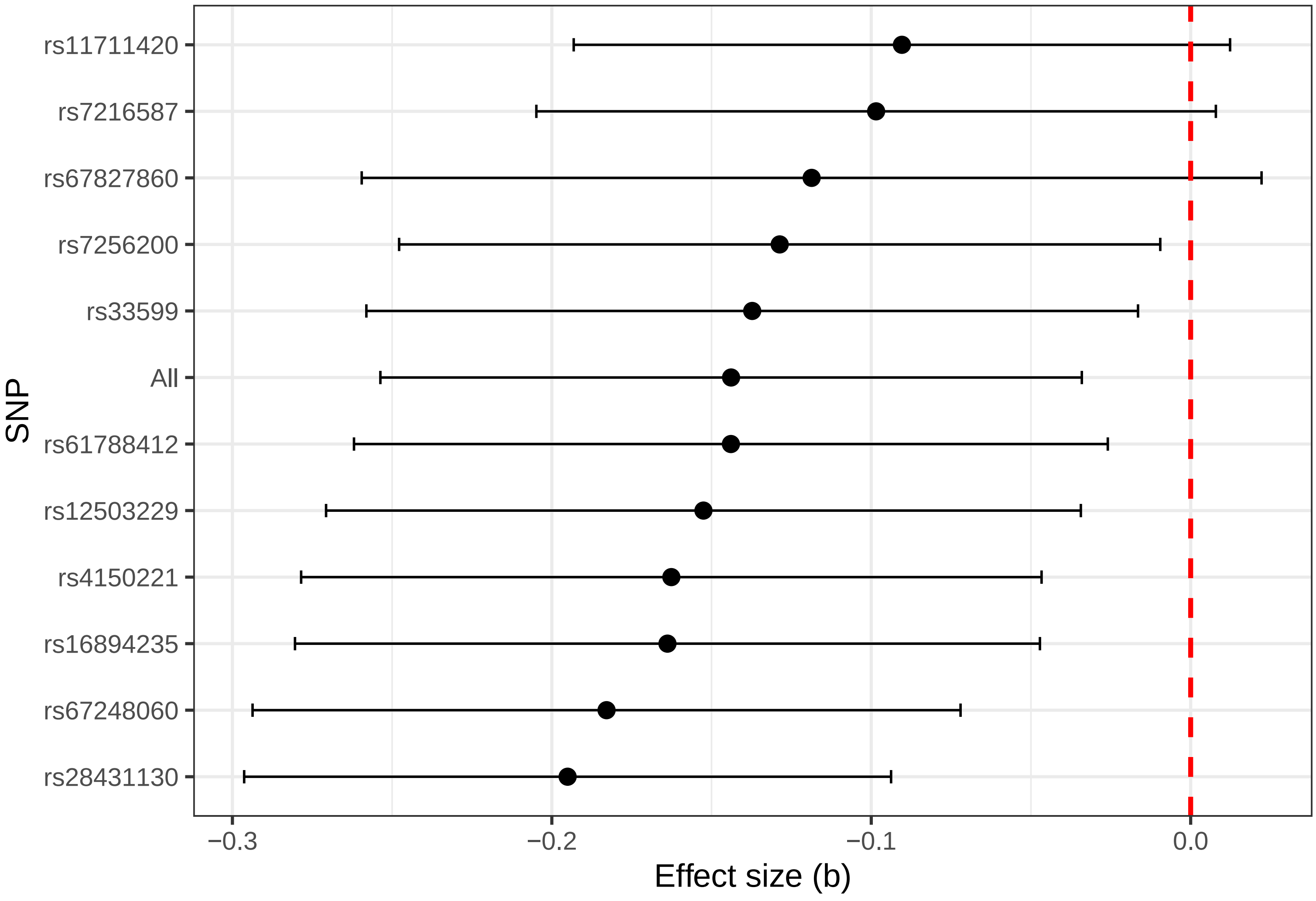

rs11711420
rs7216587
rs67827860
rs7256200
rs33599
All
rs61788412
rs12503229
rs4150221
rs16894235
rs67248060
rs28431130
SNP
−0.3
−0.2
−0.1
0.0
Effect size (b)
